# Effect of refractive error type in the amblyopic eyes on factors for treatment success in anisometropic amblyopia

**DOI:** 10.1038/s41598-021-01377-1

**Published:** 2021-11-09

**Authors:** Daye Diana Choi, Dae Hee Kim, Ungsoo Samuel Kim, Seung-Hee Baek

**Affiliations:** Department of Ophthalmology, Strabismus & Pediatric Ophthalmology Center, Kim’s Eye Hospital, 136, Yeongsin-ro, Yeongdeungpo-gu, Seoul, 07301 Korea

**Keywords:** Diseases, Medical research, Risk factors

## Abstract

To investigate the factors for treatment success in anisometropic amblyopia according to the spherical equivalent (SE) type of amblyopic eyes. Medical records of 397 children with anisometropic amblyopia aged 3 to 12 years who presented in a secondary referral eye hospital during 2010 ~ 2016 were retrospectively reviewed. Anisometropia was defined as ≥ 1 diopter (D) difference in SE, or ≥ 1.5 D difference of cylindrical error between the eyes. According to the SE of amblyopic eyes, patients were categorized into hyperopia (SE ≥ 1D), emmetropia (− 1 < SE <  + 1) and myopia (SE ≤ − 1D) groups. Treatment success was defined as achieving interocular logMAR visual acuity difference < 0.2. Multivariate logistic regression was used to analyze the factors for treatment success. Significant factors for the amblyopia treatment success in hyperopia group (n = 270) were younger age [adjusted odds ratio (aOR) (95% confidence interval, CI) = 0.529 (0.353, 0.792)], better BCVA in amblyopic eyes at presentation [aOR (95% CI) 0.004 (0, 0.096)], longer follow-up period [aOR (95%CI) = 1.098 (1.036, 1.162)], and no previous amblyopia treatment history [aOR (95% CI) 0.059 (0.010, 0.364)]. In myopia group (n = 68), younger age [aOR (95% CI) 0.440 (0.208, 0.928)] and better BCVA in amblyopic eyes [aOR (95% CI) 0.034 (0.003, 0.469)] were associated with higher odds of treatment success. There was no significant factor for treatment success in emmetropia group (n = 59) in this population. The refractive error type of amblyopic eyes at presentation affects the factors for treatment success in anisometropic amblyopia.

## Introduction

Anisometropia, the difference in refractive errors between the two eyes, is the most common cause of amblyopia in children when it is large. Amblyopia has prevalence reported as 1% to 4% in preschool-aged children^1–4^, and it may lead to irreversible vision impairment if untreated. Extensive researches have been conducted to find factors that influence the outcome of amblyopia treatment, mostly in anisometropic and/or strabismic amblyopia, and various and conflicting results have been reported^5–8^. There are also numerous reports of amblyopia treatment methods and their outcome using randomized controlled trials, however, the exact treatment method and duration vary considering each patient and guardian at physician’s discretion in the real world clinics. Even with the diversity in treatment execution, treatment of anisometropic amblyopia is successful in most cases, and there seems to be clinical characteristics of patients that lead to treatment success more easily. Our clinical experience from pediatric ophthalmology clinic in a secondary referral eye hospital led to a hypothesis that anisometropic amblyopia treatment prognosis vary depending on refractive error type in amblyopic eyes. Factors associated with the refractive error that may affect the result of anisometropic amblyopia treatment may include the spherical equivalent (SE) of the amblyopic eye, the degree of astigmatism of amblyopic eye, and/or the degree of difference in refractive errors between the two eyes. To our knowledge, there have been a few papers that attempts to analyze the factors that influence the success of amblyopia treatment according to the refractive error type of amblyopic eyes^5,9–12^. In our previous study, we found that treatment success rate was the highest, and the duration to treatment success was shortest in the emmetropia group categorized by SE of amblyopic eyes, followed by hyperopia group, and then myopia group, when each SE group demonstrated different patient chracteristics^13^. Our clinical impression was that SE type of amblyopic eye affects treatment success more than the SE difference, and hyperopic amblyopes behave differently from myopic amblyopes. In this study, we aimed to ascertain the effect of refractive error type in amblyopic eyes on the treatment success, and to find out whether the factors affecting treatment success were different according to refractive error type of amblyopic eyes in anisometropic amblyopia patient population of a usual pediatric ophthalmology practice.

## Methods

This study retrospectively reviewed medical records of patients aged 3 to 12 years who were diagnosed with anisometropic amblyopia at Strabismus & Pediatric Ophthalmology Center in Kim Eye Hospital between the period of January 1, 2010, and December 31, 2016. The patients who had any other ocular pathology or systemic condition that might affect visual acuity or those who had developmental delay were excluded from this study. Any patient with a follow-up period of less than 6 months was also excluded from this study to rule out the cases without sufficient treatment duration to reach success. Amblyopia was defined as the best corrected visual acuity (BCVA) difference of more than two logarithm of the minimum angle of resolution (logMAR) lines between the two eyes. Anisometropia was defined as spherical equivalent difference of more than 1 diopter (D), or cylinder difference of more than 1.5 D regardless of meridians, between the two eyes. Age at presentation, sex, BCVA at the first visit, refraction of both eyes at first visit, presence of manifest strabismus, previous amblyopia treatment history, BCVA at the last visit, and follow-up duration were collected retrospectively using electronic medical records. Visual acuity was measured using a Snellen chart and converted to logMAR values for analyses. Amblyopia treatment was started with a full spectacle correction after cycloplegic refraction. Patients were followed usually 1 or 2 months after spectacle prescription, and occlusion of the sound eye with patching was prescribed if there was no vision improvement in amblyopic eye. Occlusion dosage was determined considering the degree of amblyopia and response to treatment, usually from one to six hours per day. Occlusion was continued aiming for equal visual acuity on both eyes, as long as the patient and caregiver cooperate. Some of the patients were prescribed for atropine penalization (1% atropine on sound eye) instead of occlusion therapy. Subjects were followed up every 2–4 months.

Subjects were categorized according to the SE of amblyopic eye by cycloplegic refraction at the first examination, into the hyperopia group (SE ≥ 1 D), emmetropia group (− 1 < SE <  + 1), and myopia group (SE ≤ − 1 D). The treatment success was defined as achieving BCVA difference of less than two logMAR lines between the two eyes. Treatment success during the follow-up was primary outcome for univariate and multivariate logistic regression analysis. All Statistical analysis was performed using R 3.6.0 Statistical Software (R foundation for statistical computing, Vienna, Austria). To compare the three SE groups, Chi-squared test and Kruskal–Wallis test were performed using R Statistical Software. Post-hoc analysis was conducted using Mann–Whitney test, and p-values < 0.017 were considered statistically significant according to the Bonferroni’s adjustment.

Univariate and multivariate logistic regression analyses were performed to determine the influence of each factor on the success of amblyopia treatment. Factors which were statistically significant in univariate logistic regression analysis were included in multivariate logistic regression. Factors of interest (SE and cylinder of amblyopic eye) were also included in multivariate logistic regression analysis regardless of their significance in univariate analysis. Significant multicollinearity was encountered between the cylinder of the amblyopic eye and the difference of cylinder between the amblyopic eye and sound eye (Pearson’s correlation coefficient r = − 0.887, p = 0.001), and we included only cylinder of the amblyopic eye on multivariate analysis. SE difference between the two eyes at first exam were significantly correlated with BCVA of the amblyopic eye at first exam in the hyperopia and myopia group (Pearson’s correlation coefficient r = 0.425, p = 0.001 in hyperopia group, r = 0.639, p < 0.001 in myopia group, and r = 0.210, p = 0.11 in emmetropia group), therefore, we included only BCVA of amblyopic eye at first exam in multivariable analysis considering their multicollinearity. BCVA difference between two eyes at first exam and BCVA of amblyopic eye at first exam also had significant correlation (r = 0.92, p < 0.001 for hyperopia, r = 0.696, p < 0.001 for emmetropia, and r = 0.97, p < 0.001 for myopia group), and BCVA difference between two eyes at first exam was excluded from the multivariate analysis. All of the refractive errors used in this study were determined in minus cylinder form, thus an increase of cylinder value means decrease of absolute value of cylinder. This study was performed in accordance with the tenets of the Declaration of Helsinki. Approval to conduct this study was obtained from the Institutional Review Board of Kim’s Eye Hospital (IRB 2018-01-012). Informed consent was waived by the Institutional Review Board because this study was conducted retrospectively using medical records without identifiable private information.

## Results

A total of 397 children (193 male and 205 female) were included in this study. According to the SE of amblyopic eye at the first exam, there were 270 subjects (68.01%) in the hyperopia group, 59 subjects (14.86%) in the emmetropia group, and 68 subjects (17.13%) in the myopia group. Overall, for treatment of amblyopia, both glasses and patching were used in 365 (94.81%), glasses only in 20 (5.19%), and glasses and atropine penalization in 12 (3.12%) patients. Mean follow-up duration was 33.4 months (standard deviation, SD = 18.7).

Detailed demographics and clinical characteristics of the study population are shown in Table 1. Age at presentation, follow-up duration, comorbid strabismus, and proportion of patients with previous amblyopia treatment history were not significantly different among the SE groups. The emmetropia group had the smallest interocular BCVA difference and SE difference, and the largest interocular cylinder difference between the two eyes at presentation among the three SE groups. Treatment success rate during the follow-up was significantly different among the SE groups (96.61% in emmetropia group, 91.48% in hyperopia, and 82.35% in myopia group, respectively, p = 0.016 by Chi-square test, Table 1). We noticed that each SE group has different patient characteristics that may inherently related to the amblyopia treatment success, and smaller interocular BCVA difference corresponds to less severe amblyopia. We performed logistic regression analysis to compare odds of treatment success among three SE groups. After adjusting interocular BCVA difference, the adjusted odds ratio (aOR) of treatment success compared to emmetropia group were not significant in hyperopia group [aOR (95% CI) 1.21 (0.25, 5.81), p = 0.811], and in myopia group [aOR (95% CI) 0.73 (0.13, 3.99), p = 0.714].

**Table 1:** Clinical characteristics of anisometropic amblyopia patients in hyperopic, emmetropic, and myopic amblyopia groups.

	Hyperopia group (1)	Emmetropia group (2)	Myopia group (3)	p-values*	Post-hoc test
Number of patients	270	59	68		
Age at presentation (years)	5.4 ± 1.7	5.2 ± 1.4	5.0 ± 1.1	0.622^a^	
Number of male subjects (n, %)	140 (51.85)	30 (50.85)	23 (33.82)	0.070	
**Manifest strabismus (n, %)**	55 (20.37)	32 (54.24)	1 (1.5)	0.066^b^	
Esotropia	51 (18.89)	17 (28.81)	0		
Exotropia	3 (1.11)	4 (6.78)	0		
Vertical strabismus	1 (0.37)	11 (18.64)	1 (1.47)		
None	215 (79.62)	27 (45.76)	67 (98.53)		
**Previous amblyopia treatment history** Yes (n, %)	39 (14.44)	7 (11.86)	9 (13.24)	0.862^b^	
Glasses only	24 (8.89)	6 (10.17)	3 (4.41)		
Glasses + patching	14 (5.19)	–	6 (8.82)		
Glasses + atropine	1 (0.36)	1 (1.70)	–		
**Amblyopic eye at first exam**					
SE (D)	4.5 ± 1.6	0.1 ± 0.5	− 5.0 ± 3.4	** < 0.001**^**a**^	1 > 2 > 3^c^
Sph (D)	5.0 ± 1.5	1.6 ± 0.7	− 4.0 ± 3.3	** < 0.001**^**a**^	1 > 2 > 3^c^
Cyl (D)	− 1.1 ± 1.1	− 3.2 ± 1.0	− 1.9 ± 1.2	** < 0.001**^**a**^	1 > 3 > 2^c^
BCVA (logMAR)	0.6 ± 0.2	0.4 ± 0.1	0.6 ± 0.4	** < 0.001**^**a**^	1 = 3 > 2^c^
**Sound eye at first exam**					
SE (D)	1.9 ± 1.4	0.7 ± 0.6	− 0.3 ± 2.1	** < 0.001**^**a**^	1 > 2 > 3^c^
Sph (D)	2.1 ± 1.5	1.1 ± 0.8	0.1 ± 2.0	** < 0.001**^**a**^	1 > 2 > 3^c^
Cyl (D)	− 0.4 ± 1.5	− 0.8 ± 0.8	− 0.9 ± 2.0	** < 0.001**^**a**^	1 > 2 = 3^c^
BCVA (logMAR)	0.1 ± 0.1	0.1 ± 0.1	0.1 ± 0.1	0.227^a^	
ΔBCVA at first exam	0.5 ± 0.2	0.3 ± 0.1	0.6 ± 0.4	** < 0.001**^**a**^	1 = 3 > 2^c^
ΔSE at first exam (D)	2.6 ± 1.4	0.7 ± 0.5	4.6 ± 2.9	** < 0.001**^**a**^	3 > 1 > 2^c^
ΔCyl at first exam (D)	0.8 ± 0.9	2.4 ± 1.0	1.1 ± 0.9	** < 0.001**^**a**^	2 > 3 > 1^c^
**Treatment modality (n, %)**				0.217^b^	
Glasses + occlusion	249 (92.22)	55 (93.22)	61 (89.71)		
Glasses + atropine penalization	10 (3.70)	0 (0.00)	2 (2.94)		
Glasses only	11 (4.08)	4 (6.78)	5 (7.35)		
Treatment success during the follow-up (n, %)	247 (91.48)	57 (96.61)	56 (82.35)	**0.016**^**b**^	
Duration to treatment success (months)	6.0 ± 7.1	4.1 ± 4.8	6.6 ± 7.3	**0.012**^**a**^	1 = 3 > 2^c^
BCVA of amblyopic eye at treatment success (logMAR)	0.157 ± 0.07	0.16 ± 0.07	0.20 ± 0.09	0.343^a^	
Follow-up duration (months)	34.3 ± 18.5	32.6 ± 19.1	30.4 ± 19.0	0.149^a^	

Cylindrical error was described as negative values.

Numeric values are expressed as mean ± standard deviation (SD), and categorical variables are expressed as number (%).

*SE* spherical equivalent, *Sph* sphere component, *Cyl* cylinder component, *BCVA* best-corrected visual acuity, *ΔBCVA* difference of BCVA between the two eyes, *ΔSE* difference of SE between the two eyes, *ΔCyl* difference of cylinder between the two eyes.

p-values* compare hyperopia, emmetropia, and myopia groups, and p-values < 0.05 are displayed in bold.

^a^P-value using Kruskal–Wallis test.

^b^P-value by Chi-squared test.

^c^Post-hoc analysis was conducted using the Mann–Whitney test, and p-values < 0.017 were considered statistically significant according to the Bonferroni’s adjustment.

### Hyperopia group

In the univariate analysis of the hyperopia group, SE of the sound eye at the first exam, BCVA of the sound eye, and follow-up duration significantly increased the odds of treatment success, whereas age at presentation, SE of the amblyopic eye at the first exam, cylinder of the sound eye at the first exam, SE difference at the first exam, BCVA of the amblyopic eye at the first exam, BCVA difference at the first exam, manifest strabismus, and previous amblyopia treatment significantly decreased the odds of treatment success (Table 2).

**Table 2:** Crude odds ratio values of factors influencing amblyopia treatment success during follow-up in hyperopia, emmetropia, and myopia groups.

	Hyperopia group (n = 270)		Emmetropia group (n = 59)		Myopia group (n = 68)	
	Crude OR (95% CI)	P value	Crude OR (95% CI)	P value	Crude OR (95% CI)	P value
Age at presentation (years)	0.702 (0.574, 0.857)	** < 0.001**	0.114 (0.006, 2.373)	0.161	0.526 (0.299, 0.926)	**0.026**
Sex: Female vs. male	0.839 (0.356, 1.972)	0.687	0.966 (0.058, 16.200)	0.981	1.508 (0.420, 5.409)	0.529
**SE at first exam (D)**						
Amblyopic eye	0.675 (0.514, 0.885)	**0.005**	0.080 (0.001, 5.008)	0.232	1.189 (1.002, 1.411)	**0.047**
Sound eye	1.549 (1.040, 2.308)	**0.032**	89.965 (1.604, 5046.420)	**0.029**	0.847 (0.489, 1.469)	0.555
**Cylinder at first exam (D)**						
Amblyopic eye	0.855 (0.557, 1.311)	0.473	0.272 (0.078, 0.949)	**0.041**	0.642 (0.349, 1.180)	0.154
Sound eye	0.039 (0.005, 0.317)	**0.002**	0.288 (0.011, 7.576)	0.455	0.593 (0.248,1.422)	0.242
ΔSE at first exam (D)	0.450 (0.325, 0.625)	** < 0.001**	0.587 (0.065, 5.284)	0.635	0.752 (0.609,0.928)	**0.008**
Δcylinder at first exam (D)	0.914 (0.594, 1.406)	0.682	3.125 (0.546, 17.877)	0.200	1.126 (0.534,2.375)	0.755
**BCVA at first exam (logMAR)**						
Amblyopic	0.017 (0.003, 0.097)	** < 0.001**	0.027 (0.000, 17.732)	0.276	0.054 (0.008,0.367)	**0.003**
Sound	5560.108 (1.812,17,059,513.232)	**0.035**	562.179 (0.000, 1,357,708,246,281,482)	0.663	101.99 (0.047, 223,393)	0.238
ΔBCVA between the two eyes at first exam	0.011 (0.002, 0.062)	** < 0.001**	0.002 (0.000, 8.675)	0.149	0.035 (0.004, 0.272)	**0.001**
Follow-up duration (months)	1.069 (1.028, 1.111)	** < 0.001**	0.951 (0.890, 1.016)	0.135	1.014 (0.978,1.051)	0.456
Presence of manifest strabismus (yes vs. no)	0.399 (0.166, 0.957)	**0.040**	0.118 (0.006, 2.134)	0.148	1.222 (0.234,6.397)	0.812
**Treatment modality: (ref = glasses only)**						
Glasses + Patching	2.413 (0.489, 11.903)	0.279	0.000 (0.000, Inf)	0.996	1.136 (0.115,11.182)	0.913
Glasses + Atropine penalization	9,454,402.746 (0.000, Inf)	0.990			3,912,840.198 (0.000,Inf)	0.993
Previous amblyopia treatment history (yes vs. no)	0.215 (0.086, 0.540)	**0.001**	0.118 (0.006, 2.134)	0.148	1.833 (0.207, 16.213)	0.586

Cylindrical error was described as negative values. Numeric values are expressed as mean ± SD, and categorical variables are expressed as number (%).

*OR* odds ratio, *CI* confidence interval, *D* diopter, *SE* spherical equivalent; *BCVA* best-corrected visual acuity, *inf* infinity, *ref* reference level, *ΔSE* difference of SE between the two eyes, *ΔCyl* difference of cylinder between the two eyes.

*P-value < 0.05 is considered statistically significant and displayed in bold.

In the multivariate analysis, follow-up duration [aOR (95% CI) 1.098 (1.036, 1.162)] significantly increased the odds of treatment success. On the contrary, age at presentation [aOR (95% CI) 0.529 (0.353, 0.792)], larger logMAR BCVA of amblyopic eye at the first exam [aOR (95% CI) 0.004 (0, 0.096)], and history of previous amblyopia treatment [aOR (95% CI) 0.059 (0.011, 0.364)] significantly decreased the odds of treatment success (Table 3). SE of the amblyopic eye and the sound eye at the first exam, a cylinder of amblyopic eye at the first exam, and BCVA of the sound eye at presentation were not significant factors in multivariate analysis of the hyperopia group. In a separate multivariate analysis including the SE difference between the two eyes at first exam instead of the BCVA of amblyopic eye at first exam considering multicollinearity, the SE difference was not a significant factor for the treatment success (data not shown).

**Table 3:** Adjusted odds ratio values of factors influencing amblyopia treatment success during follow-up in hyperopia, emmetropia, and myopia groups.

	Hyperopia group (n = 270)^a^		Emmetropia group (n = 59)^b^		Myopia group (n = 68)^c^	
	aOR (95% CI)	P value	aOR (95% CI)	P value	aOR (95% CI)	P value
Age at presentation (years)	0.529 (0.353, 0.792)	**0.002**	0.000 (0.000, Inf)	0.998	0.440 (0.208, 0.928)	**0.031**
**SE at first exam (D)**						
Amblyopic eye	0.690 (0.369,1.291)	0.246	0.000 (0.000, Inf)	0.996	1.002 (0.722,1.391)	0.988
Sound eye	1.779 (0.853, 3.710)	0.125	6.137 (0.000, Inf)	0.996		
**Cylinder at first exam (D)**						
Amblyopic eye	0.946 (0.364, 2.461)	0.910	0.000 (0.000, Inf)	0.996	0.430 (0.167, 1.104)	0.079
Sound eye	0.053 (0.003, 1.065)	0.055				
**BCVA at first exam (logMAR)**						
Amblyopic eye	0.004 (0.000, 0.096)	** < 0.001**			0.034 (0.003, 0.469)	**0.012**
Sound eye	1.347 (0.000, 119,854.800)	0.959				
Follow-up duration (months)	1.098 (1.036, 1.162)	**0.001**				
Presence of manifest strabismus (yes vs. no)	2.185 (0.502, 9.514)	0.298				
Previous amblyopia treatment history (yes vs. no	0.059 (0.010, 0.364)	**0.002**				

*aOR* adjusted odds ratio, *CI* confidence interval, *D* diopter, *SE* spherical equivalent, *BCVA* best-corrected visual acuity, *Inf* infinity.

^a^Multivariate model of the hyperopia group started with age at presentation, SE of amblyopic eye, SE of sound eye, cylinder of amblyopic eye, cylinder of sound eye, logMAR BCVA of amblyopic eye, and logMAR BCVA of sound eye at first exam, follow-up duration, presence of manifest strabismus, and previous amblyopia treatment history.

^b^Multivariate model of the emmetropia group started with age at presentation, SE of amblyopic eye, SE of sound eye, and cylinder of amblyopic eye.

^c^Multivariate model of the myopia group started with age at presentation, SE of amblyopic eye, cylinder of amblyopic eye, and logMAR BCVA of the amblyopic eye at first exam.

### Emmetropia group

In the univariate analysis of the emmetropia group, larger SE of the sound eye at the first exam [crude OR (95%CI) = 89.965 (1.604, 5046.420)] significantly increased the odds of treatment success, but larger negative cylinder of the amblyopic eye at the first exam [crude OR (95%CI) = 0.272 (0.078, 0.949)] significantly decreased the odds of treatment success (Table 2). However, both were not significant factors in multivariate analysis in this group (Table 3).

### Myopia group

Univariate analysis of the myopia group found that the SE of amblyopic eye at the first exam significantly increased the odds of treatment success, whereas age at presentation, SE difference at the first exam, and BCVA of the amblyopic eye at the first exam decreased the odds of treatment success (Table 2). However, SE of the amblyopic eye at the first exam and SE difference at the first exam were not significant factors when considering other factors in multivariate analysis. On multivariate analysis, only older age at presentation [aOR (95%CI) = 0.440 (0.208, 0.928)] and higher logMAR BCVA of amblyopic eye at the first exam [aOR (95%CI) = 0.034 (0.003, 0.469)] significantly decreased the odds of treatment success in the myopia group (Table 3).

## Discussion

In the present study, 397 patients who were treated > 6 months for anisometropic amblyopia in a secondary referral eye hospital were analyzed. The factors affecting treatment success in anisometropic amblyopia were different according to the SE type of the amblyopic eyes. The age and the BCVA of the amblyopic eye at presentation were factors affecting the odds of amblyopia treatment success in hyperopia and myopia groups. Longer follow-up period, and the absence of previous treatment history were significant factors increasing the odds of treatment success only in hyperopia group. Furthermore, there was no significant factor affecting treatment success in the emmetropia group.

There are diverse reports about treatment outcome of anisometropic amblyopia. However, as the hyperopic difference in refractive error are more amblyogenic than myopic differences^14^, many anisometropic amblyopia studies were comprised of mostly hyperopic patients^6,15,16^. Small number of papers have analyzed treatment outcome according to refractive error type of amblyopic eye. Keech et.al. reported that the refractive error type of anisometropia was significant factor affecting anisometropic amblyopia treatment outcome as patients with myopic and compound myopic/mixed astigmatism had poorer visual outcomes than hyperopia^7^. On the contrary, Meenakshi et al. found that greatest amount of improvement in visual acuity was seen in myopic anisometropic patients and the least in hyperopes^5^. Husseint et al. reported that the refractive error type of amblyopia was not a significant factor for amblyopia treatment outcome^15^. Our study classified anisometropic amblyopia as hyperopia, emmetropia, and myopia groups according to SE of amblyopic eye at first exam and analyzed the factors for treatment success.

Treatment success rates during the follow-up were the highest in emmetropia group, followed by hyperopia group and then myopia group. This result is in accordance to our previous study^13^, even though this study included only the patients with sufficient follow-up duration of more than six months, considering the duration for treatment success in our previous study (mean 6.0, 4.1, and 6.6 months for the hyperopia, emmetropia, and myopia group). However, the interocular BCVA difference at presentation, which reflects the severity of amblyopia, was significantly smaller in emmetropia group than in hyperopia or myopia group. After adjusting the interocular BCVA difference, the adjusted odd ratios of treatment success compared to emmetropia group were not significant in hyperopia group and in myopia group, and it may be interpreted as that odds of treatment success would not be different among SE groups if they are similar in amblyopia severity. However, interocular BCVA difference at presentation may reflect the characteristics of each SE group, considering we included all subjects satisfying inclusion criteria during study period. In that manner, clinician may expect that if the SE of amblyopic eye is close to emmetropia, the interocular BCVA difference may be smaller, and the probability of treatment success can be higher.

In our univariate analyses results, the significance of SE in amblyopic eye, cylinder of the amblyopic eye, and SE difference were different in each SE group. In univariate analyses of hyperopia group, SE of amblyopic eye, SE of sound eye, cylinder of sound eye, and difference of SE between two eyes at first exam were significant factors. In the univariate analyses of emmetropia group, SE of sound eye and cylinder of amblyopic eye, while in the myopia group, SE of amblyopic eye and difference of SE between two eyes were significant factors. However, the significance disappeared as the other factors were adjusted in each SE group, which implies that other factors unrelated to refractive error were more important for treatment success than the refractive error itself within each SE group.

Previously, Hussein et al. reviewed the record of 104 children aged 3 to 8 years with anisometropic amblyopia, and found that neither the type or amount of refractive error nor the difference in the refractive power between the two eyes was a significant risk factor for treatment failure^15^. Myopic anisometropia were only 22% in their study population, and rest were classified as hyperopic anisometropia. They reported age above 6 at the onset of treatment, and worse than 20/200 initial BCVA of amblyopic eye as failure risk factors. These results are in accordance with our negative results about refractive factors, also with the age and BCVA of amblyopic eye at presentation as significant factors for treatment success in hyperopia and myopia groups. On the contrary, Cobb et al. reviewed 112 children with anisometropic amblyopia who treated with spectacles and patching, and reported that the age at presentation had no effect on the final visual outcome, while the amount of refractive error and degree of anisometropia do correlate strongly with final visual acuity^16^. It is notable that most (87%) of their study population were hyperopic and the myopic anisometropia were only 12.5%. Kirandi et al. reviewed 64 children aged 7–9 years with anisometropic amblyopia who were treated with spectacles and patching, and reported that refractive error of SE >  + 3D in the amblyopic eye was a risk factor for treatment failure^6^. The study population of Kirandi et al. was also mostly comprised of hyperopic anisometropic subjects (n = 60, 93.7%). The results of Cobb et al. and Kirandi et al. may be comparable to our results of hyperopia group. In our hyperopia group, however, the age at presentation was a significant factor, and neither the amount of SE nor the difference of SE were a significant factor for treatment success. These different results may be due to different definition of treatment outcome, and method of analyses. Our study has its merits to analyze the effect of SE amount or the SE difference on the amblyopia treatment success in the hyperopia group, and adjusted factors other than refractive errors, such as follow-up duration or concomitant strabismus. Pang et al. prospectively analyzed myopic anisometropic amblyopia patients, and found that the final VA in the amblyopic eye was associated with the VA in the amblyopic eye at baseline and the amount of anisometropia^10^. The improvement in VA with patching was inversely associated with patients age. In the similar manner, the age at presentation and the VA of the amblyopic eye at first exam were significant factor for treatment success in our myopia group. However, the difference of SE was not a significant factor in our study.

In this study, the cylinder value of the amblyopic eye was not a significant factor for treatment success in multivariate analyses in all SE groups. The emmetropia group had a larger cylinder value than those of the hyperopia and myopia group. The emmetropia group subjects in this study may have been classified as astigmatic or mixed astigmatic amblyopia in other studies. There are conflicting studies on the impact of astigmatism for the treatment success in amblyopia. Hussein et al. reported that eyes with significant astigmatism were less likely to achieve successful outcomes in cases of anisometropic amblyopia^17^, while others reported that the degree of astigmatism is not a significant factor for treatment outcome^6,18^.

Age at presentation was a significant factor for amblyopia treatment success in hyperopia and myopia group of our study. The younger at presentation, the more likely it was to have the greater odds of treatment success in anisometropic amblyopia. This result agrees with many previous reports, which have found the better visual outcomes in younger than older patients^9,15,19,20^. However, some authors insisted that the age at presentation had no effect on the final visual outcome^7,16,21^. These various results might be due to the different definition of treatment success. It is notable that the age at presentation was not a significant factor for treatment success in our emmetropia group. One study about astigmatic amblyopia reported that the age at presentation did not influence final visual acuity^18^. Even though our emmetropia group may comparable with usual astigmatic anisometropia group from other study due to its large cylinder value, but our emmetropia group only included subjects who had SE is close to emmetropia. We may speculate that emmetropic SE is a strong prognostic factor that can overcome the age.

BCVA of amblyopic eye at the first examination was also a significant factor in both the hyperopia and myopia groups, and larger LogMAR value, which is worse BCVA at presentation, significantly decreased the odds of treatment success. These results are consistent with those of previous studies^7,17–19^. On the contrary, in the emmetropia group, BCVA of the amblyopic eye was not a significant factor affecting treatment success. This result could be due to the fact the initial BCVA of the amblyopic eye in emmetropia group was better than the other two SE groups. It is worthy noted that there was a significant correlation between BCVA of amblyopic eye and interocular SE difference at first examination. Due to this significant correlation, we had to exclude SE difference from multivariate analyses. The interocular SE difference itself was not significant factor in all groups in the analysis including SE difference instead of BCVA of amblyopic eye (data not shown). Even though, clinicians should pay attention to initial SE difference as well as initial BCVA of amblyopic eye due to their close relationship.

Follow-up duration had a significant positive effect on treatment success in the hyperopia group only. The longer follow-up duration made the odds of treatment success increase by 1.1 times. It can be suspected that the longer follow-up duration is associated with good compliance and selection bias, therefore yield good treatment results. However, follow-up duration is not a significant factor for the emmetropia and myopia groups whereas their follow-up durations were not significantly different, therefore different SE group demonstrated different treatment response by the follow-up duration.

Patients with history of previous amblyopia treatment showed significantly lower odds of treatment success in hyperopia group. This is in accordance with previous studies which found history of amblyopia treatment as a risk factor for treatment failure^22,23^. As with many amblyopia studies, the patient population of the those papers is mostly comprised of hyperopic amblyopia. The history of previous amblyopia treatment was not significant factor for emmetropia and myopia group in our result. However, both group had relatively small number of subjects compared to hyperopia group, and further study with more subjects is needed to clarify this.

The results that there were no significant factors related to treatment success in the emmetropia group might need some interpretation. We speculated that the relatively small number of patients with high success rate made lack of diversity to predict success and failure in this group. Further study with larger number of subject is warranted to find the significant factor for emmetropia group.

We focused on achieving treatment success any time during the follow-up, not the success at the last visit in our study. Defining treatment success at the last visit will reflect fluctuation of visual acuity such as recurrence with or without recovery after recurrence. In our study, the treatment success rate during the follow-up and at the last exam were not significantly different within each SE group (p = 0.055, 1.0, 0.519 for the hyperopia, emmetropia, and myopia group respectively, p-values by Chi-squared test, data not shown). There were 14 patients (5.19%) in the hyperopia group, and 3 patients (4.41%) in the myopia group who had once achieved treatment success, but failed to maintain it at the last exam. In a prospective follow-up study after treatment cessation in children with successfully treated amblyopia due to anisometropia, strabismus or both, the risk of amblyopia recurrence was reported to be 24% within the first year off treatment^24^. In other retrospective study also reported recurrence of amblyopia after a cessation of occlusion therapy was 27% within the first year off treatment^25^. Relatively lower recurrence rate of our result than previous studies may be due to given active treatment during the whole follow-up period in our subjects, such as re-occlusion therapy. It is also interesting that the emmetropia group had no recurrence at all, while the other two SE groups had some. Due to the small number of recurrent cases and possibility of visual acuity fluctuation during the follow-up, we defined our primary end-point as the treatment success rate during the follow-up.

There are some limitations in our study. Due to the retrospective study design, treatment method and duration were not able to controlled. Also, there was a limitation to evaluate the compliance of patching and wearing glasses. Lastly, due to relatively high success rates, especially in emmetropia group, power to find significant factor for treatment success may have been limited. However, this study has its merit that a large scale study population recruited from a secondary referral eye hospital which would reflect the general population better than the tertiary referral hospitals. The goal of this study was to compare the treatment outcome depending on the SE type of amblyopic eye in anisometropia amblyopia in real world pediatric ophthalmology clinic. Also this is one of a few studies to evaluate the factors affecting amblyopia treatment success according to the refractive error type of amblyopic eye.

## Conclusions

In conclusion, treatment success rates were different by SE type of the amblyopic eyes in this anisometropic amblyopia population from a secondary referral eye hospital, with different baseline patient characteristics in each SE group. Refractive error-related factors including the amount of SE and astigmatism of the amblyopic eyes, and the difference in refractive errors between the two eyes were not significant factors affecting the amblyopia treatment success after adjusting other factors in all SE groups, However, the factors for treatment success were different according to the refractive error type of amblyopic eyes.

## References

[1] Dirani M, Zhou B, Hornbeak D (2010). Prevalence and causes of decreased visual acuity in Singaporean Chinese preschoolers. Br. J. Ophthalmol..

[2] Williams C, Northstone K, Howard M (2008). Prevalence and risk factors for common vision problems in children: Data from the ALSPAC study. Br. J. Ophthalmol..

[3] Multi-ethnic Pediatric Eye Disease Study G (2008). Prevalence of amblyopia and strabismus in African American and Hispanic children ages 6 to 72 months the multi-ethnic pediatric eye disease study. Ophthalmology.

[4] Friedman DS, Repka MX, Katz J (2009). Prevalence of amblyopia and strabismus in white and African American children aged 6 through 71 months the Baltimore Pediatric Eye Disease Study. Ophthalmology.

[5] Karthikeyan B, Meenakshi S (2009). The results of treatment of anisomyopic and anisohypermetropic amblyopia. Int. Ophthalmol..

[6] Kirandi EU, Akar S, Gokyigit B (2017). Risk factors for treatment failure and recurrence of anisometropic amblyopia. Int. Ophthalmol..

[7] Kutschke PJ, Scott WE, Keech RV (1991). Anisometropic amblyopia. Ophthalmology.

[8] Rosenthal AR, von Noorden GK (1971). Clinical findings and therapy in unilateral high myopia associated with amblyopia. Am. J. Ophthalmol..

[9] Curtin BJ, Schlossman A (1976). Unilateral high myopia in childhood: Clinical characteristics and treatment. Am. Orthop. J..

[10] Pang Y, Allison C, Frantz KA (2012). A prospective pilot study of treatment outcomes for amblyopia associated with myopic anisometropia. Arch.. Ophthalmol..

[11] Pang Y, Frantz KA, Block S (2015). Effect of amblyopia treatment on macular thickness in eyes with myopic anisometropic amblyopia. Invest. Ophthalmol. Vis. Sci..

[12] Priestley BS, Hermann JS, Bloom M (1963). Amblyopia secondary to unilateral high myopia* Results of pleoptic therapy *From the Department of Pleoptics, New York Eye and Ear Infirmary. This study was supported by a Fight-for-Sight grant-in-aid of the National Council to Combat Blindness Inc., New York. Am. J. Ophthalmol..

[13] Cho S-Y, Kim DH, Kim US, Baek S-H (2020). Anisometropic amblyopia: Distribution of refractive errors and clinical characteristics of patients from an eye hospital. Ann. Optom. Contact Lens.

[14] Jampolsky A, Flom BC, Weymouth FW, Moses LE (1955). Unequal corrected visual acuity as related to anisometropia. AMA Arch. Ophthalmol..

[15] Hussein MAW, Coats DK, Muthialu A (2004). Risk factors for treatment failure of anisometropic amblyopia. J. Am. Assoc. Pediatr. Ophthalmol. Strabismus.

[16] Cobb CJ, Russell K, Cox A, MacEwen CJ (2002). Factors influencing visual outcome in anisometropic amblyopes. Br. J. Ophthalmol..

[17] Hussein MA, Coats DK, Muthialu A (2004). Risk factors for treatment failure of anisometropic amblyopia. J. AAPOS..

[18] Lee HC, Kim MM (1996). Visual prognosis in children with astigmatic amblyopia. J. Korean Opthalmol. Soc..

[19] Kivlin JD, Flynn JT (1981). Therapy of anisometropic amblyopia. J. Pediatr. Ophthalmol. Strabismus..

[20] Sullivan M (1976). Results in the treatment of anisometropic amblyopia. Am. Orthop. J..

[21] Hardman Lea SJ, Loades J, Rubinstein MP (1989). The sensitive period for anisometropic amblyopia. Eye.

[22] Scheiman MM, Hertle RW, Beck RW (2005). Randomized trial of treatment of amblyopia in children aged 7 to 17 years. Arch. Ophthalmol..

[23] Holmes JM, Lazar EL, Melia BM (2011). Effect of age on response to amblyopia treatment in children. Arch. Ophthalmol..

[24] Holmes JM, Melia M, Bradfield YS, Cruz OA, Forbes B, Pediatric Eye Disease Investigator Group (2007). Factors associated with recurrence of amblyopia on cessation of patching. Ophthalmology.

[25] Bhola R, Keech RV, Kutschke P (2006). Recurrence of amblyopia after occlusion therapy. Ophthalmology.

